# Irrigation and Nitrogen Regimes Promote the Use of Soil Water and Nitrate Nitrogen from Deep Soil Layers by Regulating Root Growth in Wheat

**DOI:** 10.3389/fpls.2018.00032

**Published:** 2018-02-01

**Authors:** Weixing Liu, Geng Ma, Chenyang Wang, Jiarui Wang, Hongfang Lu, Shasha Li, Wei Feng, Yingxin Xie, Dongyun Ma, Guozhang Kang

**Affiliations:** ^1^College of Agronomy, Henan Agricultural University, Zhengzhou, China; ^2^State Key Laboratory of Wheat and Maize Crop Science, Henan Agricultural University, Zhengzhou, China; ^3^National Engineering Research Centre for Wheat, Henan Agricultural University, Zhengzhou, China

**Keywords:** irrigation regime, nitrogen regime, root length density, grain yield, water use, soil NO_3_-N content

## Abstract

Unreasonably high irrigation levels and excessive nitrogen (N) supplementation are common occurrences in the North China Plain that affect winter wheat production. Therefore, a 6-yr-long stationary field experiment was conducted to investigate the effects of irrigation and N regimes on root development and their relationship with soil water and N use in different soil layers. Compared to the non-irrigated treatment (W0), a single irrigation at jointing (W1) significantly increased yield by 3.6–45.6%. With increases in water (W2, a second irrigation at flowering), grain yield was significantly improved by 14.1–45.3% compared to the W1 treatments during the drier growing seasons (2010–2011, 2012–2013, and 2015–2016). However, under sufficient pre-sowing soil moisture conditions, grain yield was not increased, and water use efficiency (WUE) decreased significantly in the W2 treatments during normal precipitation seasons (2011–2012, 2013–2014, and 2014–2015). Irrigating the soil twice inhibited root growth into the deeper soil depth profiles and thus weakened the utilization of soil water and NO_3_-N from the deep soil layers. N applications increased yield by 19.1–64.5%, with a corresponding increase in WUE of 66.9–83.9% compared to the no-N treatment (N0). However, there was no further increase in grain yield and the WUE response when N rates exceeded 240 and 180 kg N ha^−1^, respectively. A N application rate of 240 kg ha^−1^ facilitated root growth in the deep soil layers, which was conducive to utilization of soil water and NO_3_-N and also in reducing the residual NO_3_-N. Correlation analysis indicated that the grain yield was significantly positively correlated with soil water storage (SWS) and nitrate nitrogen accumulation (SNA) prior to sowing. Therefore, N rates of 180–240 kg ha^−1^ with two irrigations can reduce the risk of yield loss that occurs due to reduced precipitation during the wheat growing seasons, while under better soil moisture conditions, a single irrigation at jointing was effective and more economical.

## Introduction

The North China Plain covers an area of 3.2 × 10^5^ km^2^. Due to climatic conditions, a highly intensive winter wheat and summer maize double-cropping system is the dominant, high-yielding agricultural production system (Zhao et al., 2007), supplying more than 50% of the winter wheat (*Triticum aestivum* L.) and 33% of the summer maize (*Zea mays* L.) produced in China (National Bureau of Statistics, 2013). The crop rotation system consumes 800–850 mm of water annually (about 450 mm for winter wheat and 360 mm for summer maize). However, annual precipitation in the region ranges from 500 to 600 mm, with an average of 200–220 mm falling during the wheat growing season (Liu et al., 2001; Yang et al., 2015). Water is the main limiting factor in the North China Plain, and crop production relies mainly on groundwater irrigation (Hu et al., 2010; Zhang et al., 2010; Li et al., 2015). As a result, groundwater tables are dropping ~1.5 m per year, which is threatening the development of sustainable agriculture (Fang et al., 2010; Li et al., 2015). In general, there are 4–5 irrigations supplied by some farmers during the wheat growing seasons in this region, which leads to a lower water use efficiency (WUE; Sun et al., 2010). Appropriate irrigation regimes are therefore fundamental to increase WUE so as to develop sustainable wheat production in the North China Plain (Li et al., 2015). Current evidence suggests that with deficit irrigation, nearly 22% of irrigation water might be saved without any significant reduction in wheat yield (Hamid et al., 2010; Shaughnessy et al., 2012; Li et al., 2015). Therefore, it is important to obtain a balance between high yield and reduced water supply in winter wheat by increasing its WUE in this region (Zhong and Shangguan, 2014).

Nitrogen (N) is fundamental plant nutrient that affects productivity and WUE in wheat (Zhou et al., 2011; Devadas et al., 2014; Wang et al., 2015) and improves soil fertility (Hai et al., 2010). The increasing use of N fertilizer has been shown to significantly increase grain yields by 50–60% in the dry-land of the Loess Plateau (Fan et al., 2005) and by 20–80% in a rainfed Mediterranean environment (Bassoa et al., 2010). Zhong and Shangguan (2014) reported that the wheat grain yield and WUE increased as the N rate increased from 0 to 270 kg N ha^−1^, while excessive N application had no positive effect on either parameter. However, a large range of N application rates exists in the North China Plain, some of which exceed 300 kg N ha^−1^. In fact, excessive N application not only causes a waste of resources and economic losses, but also has an adverse effect on wheat yield and the environment (Godfray et al., 2010; Hvistendahl, 2010). In the North China Plain, 221–620 kg N ha^−1^ of residual nitrate was detected in the 0–1.0 m soil profile at N application rates of 200–300 kg N ha^−1^ after two wheat-maize rotations (Fang et al., 2006). Therefore, it is highly important to use water and N fertilizer in a reasonable manner in order to reduce the cost of resources in this region.

Root distribution is often utilized in the analysis of soil-root-shoot-atmosphere interactions (Smit et al., 1994; Asseng et al., 1997; Dai et al., 2014). The temporal and spatial distribution of the wheat root system affects potential water and nutrient absorption (Hund et al., 2009; Dai et al., 2014; Xu et al., 2016). Irrigation and nitrogen regimes affect the root distribution in wheat, which strongly influences soil water consumption (Zhang et al., 2009; Wang et al., 2014) and soil NO_3_-N content (Xu et al., 2016). In general, slight water shortages and nitrogen deficiency during vegetative growth of wheat cause an increase in vertical root penetration, decreasing the root length density (RLD) in the top soil layer and increasing the RLD in deeper layers (Li et al., 2010; Wang et al., 2014; Xu et al., 2016). Roots in the deep soil layers (>0.8 m) are essential for nutrient and moisture absorption and, therefore, growth and final yield in wheat (Dai et al., 2014; Xu et al., 2016).

Although some studies have been conducted on root systems, water-N use, and yield under different fertilization and crop management schemes (Zhou et al., 2011; Shen et al., 2012; Chu et al., 2016), fixed–position experiments on irrigation and nitrogen regimes in wheat-maize fields have been relatively poorly studied in the North China Plain, especially under high yielding conditions. In addition, the relationships between root growth and the use of soil water and N in different soil layers remain to be further investigated. Moreover, the yield and WUE in wheat is also influenced by meteorological factors and seasonal variation (Kang et al., 2002). Therefore, a 6-yr-long stationary field experiment was initiated in 2010 in the western part of the North China Plain. The objectives of the present investigation were as follows: (1) to examine the impacts of irrigation regimes and N application rates on wheat grain yield; (2) to establish relationships between root development and soil water reduction amount and NO_3_-N content at different soil depths, and (3) to determine the optimal combination of irrigation and nitrogen application rate for winter wheat in the North China Plain.

## Materials and methods

### Experimental site

**Figure 1:**
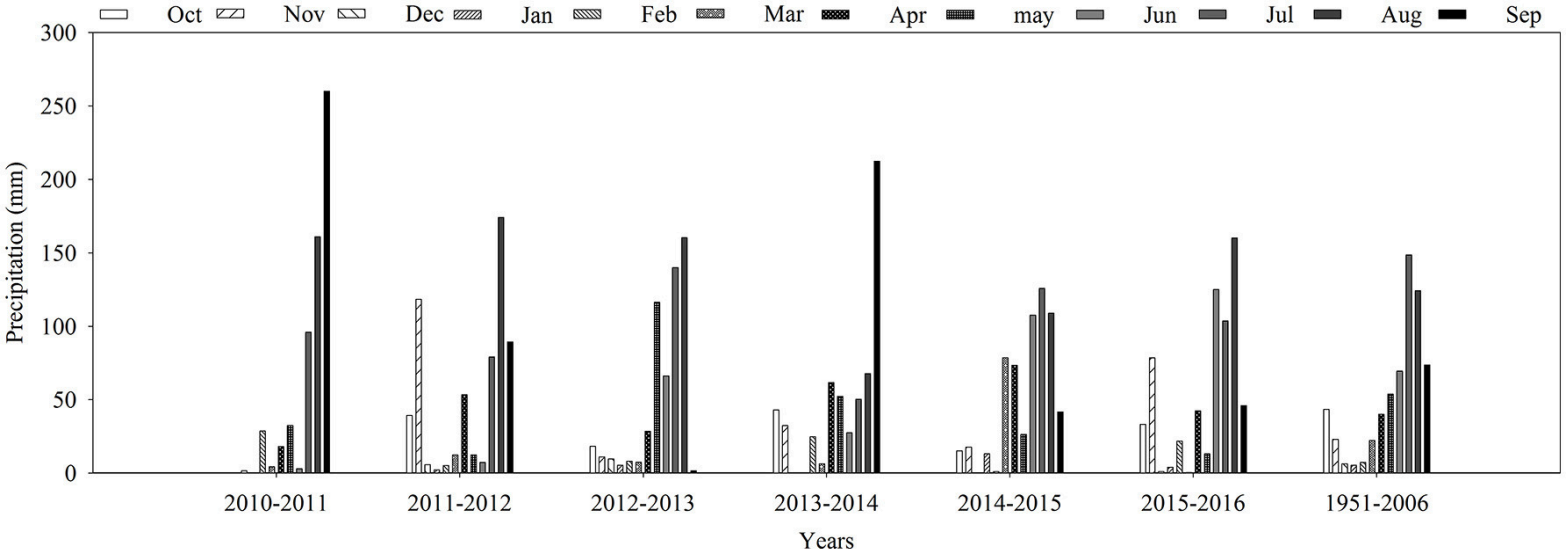
Annual precipitation by month (October to September) during the six wheat-maize growing seasons from 2010–2011 to 2015–2016 and the corresponding monthly averages from 1951 to 2006 at the experimental site.

The field experiments were performed in six winter wheat-summer maize rotations from 2010 to 2016 at Henan Agricultural University Experimental Station, located in the west of the North China Plain (113°38′39″ E, 34°47′51″ N). The soil is an alluvial soil (Gong et al., 2007), belonging to the Ochri-Aquic Cambosols (32.3% sand, 57.7% silt, 10.0% clay). In the cultivated layer (0.2 m), the pH was 7.82 with a field capacity of 23.9% volumetric soil water and a bulk density of 1.28 g m^−3^. The amounts of organic matter, total N, available phosphorus and available potassium were 17.47 g kg^−1^, 0.84 g kg^−1^, 18.83 mg kg^−1^, and 252.56 mg kg^−1^, respectively. The experimental site is characterized by a north temperate continental monsoon climate zone with an annual mean temperature of 14.3°C and annual sunshine duration of 2300 h. The average (1951–2006) annual rainfall is 617.1 mm, with the majority (60–75%) occurring in the summer months (June to September), and an average of 201.2 mm falling during the wheat growing season. Precipitation during the experimental seasons for wheat and maize is shown in Figure 1. Relatively dry weather prevailed during the wheat growing seasons of 2010–2011, 2012–2013, and 2015–2016; rainfall was 84.8, 183.9, and 194.3 mm, respectively, compared to the long-term average of 201.2 mm (1951–2006). However, the rainfall pattern showed monthly variability. During the 2012–2013 and 2015–2016 wheat growing seasons, there was only 13.1 and 7.9 mm of rainfall from jointing to flowering (March 10 to April 15), which was a major limiting factor to wheat production (Xue et al., 2006; Karam et al., 2009).

### Experimental design and crop management

The experiment, a typical winter wheat-summer maize rotation, started with the winter wheat season of October 2010, and ended after the winter wheat harvest in June 2016, covering 11 successive seasonal crops in the same experimental plot. Each experimental plot was 7 m in length and 2.9 m in width. The plots were arranged in a split-plot design with four replicates. The main plots were assigned to three irrigation regimes: no irrigation water applied after sowing (W0), a single irrigation at jointing (W1), and two irrigations, one each at jointing and flowering (W2) during the wheat growing seasons. During the maize growing seasons, in all treatments irrigation was provided based on critical growth stages, such as the V12 stage, tasseling stage, and elongation stage during the 2012, 2013, and 2014 maize growing seasons, respectively (Table 1). Irrigation was applied by a movable sprinkler system, and 75 mm was applied each time as measured by a water meter. The treatment details are given in Table 1. Subplots were treated with five nitrogen rates: 0, 90, 180, 240, and 300 kg N ha^−1^ during wheat growing season, and 0, 112.5, 225, 300, and 375 kg N ha^−1^ during the maize growing season; these are referred to as N0, N1, N2, N3, and N4, respectively.

**Table 1:** Water supply for the 2010–2011 to 2015–2016 winter wheat and summer maize growing seasons.

**Crop**	**Water source**		**2010–2011**	**2011–2012**	**2012–2013**	**2013–2014**	**2014–2015**	**2015–2016**
Wheat	Rainfall (mm)		84.8	236.1	183.9	220.4	222.7	194.3
	Irrigation (mm)	W0	0	0	0	50	0	0
		W1	75	105	75	125	75	75
		W2	150	150	150	200	150	150
	Total (mm)	W0	84.8	236.1	183.9	270.4	222.7	194.3
		W1	159.8	341.1	258.9	295.4	297.7	269.3
		W2	234.8	386.1	333.9	420.4	372.7	344.3
Maize	Rainfall (mm)		520.0	362.2	388.4	357.8	386.5	434.7
	Irrigation (mm)	W0	75	0	75	75	75	75
		W1	75	60	75	150	75	75
		W2	75	60	150	150	75	75
	Total (mm)	W0	595.0	362.2	463.4	432.8	461.5	509.7
		W1	595.0	422.2	463.4	507.8	461.5	509.7
		W2	595.0	422.2	538.4	507.8	461.5	509.7

*Irrigation in the W0 treatment was followed by post-sowing irrigation to all plots regardless of treatment to ensure proper germination and establishment of the seedlings. Irrigation was applied at the V12, tasseling (VT), and V6 stage during the 2012, 2013, and 2014 maize growing seasons, respectively*.

Wheat (cv. “Aikang 58” until 2012, then “Yumai 49–198”) was sown on 12 October each year at a sowing rate of 225 kg ha^−1^ with 0.2 m row spacing. The wheat was harvested in late May-early June of the following year. Phosphorus was applied as triple superphosphate (P_2_O_5_; 46%) at a rate of 150 kg P ha^−1^, and potash was applied as potassium chloride (K_2_O; 46%) at a rate of 100 kg K ha^−1^. Half the amount of nitrogen (urea; 46% N), and all phosphorus and phosphate was spread uniformly over the soil surface and then drilled into the upper 0.15 m at sowing. The other half of the nitrogen was applied at the jointing stage for each treatment. Maize (cv. “Zhengdan 958”) was planted by hand immediately after the wheat harvest in the same plots. P_2_O_5_ was applied at 90 kg ha^−1^, K_2_O at 120 kg ha^−1^, and 30% of the nitrogen for each treatment was applied at the elongation stage of maize, with the remaining 70% applied at the V12 stage.

Additional protective measures were taken to ensure healthy growth such as the spray application of insecticides, fungicides, and herbicides. Accordingly, no significant incidence of pests, diseases, or weeds was observed in any treatment during any of the growing seasons.

### Soil samples and analysis

Soil cores were collected every 0.2 m to a final depth of 1.6 m using a soil auger (0.05 m diameter) before sowing and after harvest in all experimental plots. Two cores were collected from each plot. Samples were then analyzed for soil NO_3_-N content (SNC; mg kg^−1^). Briefly, samples of fresh soil were extracted by shaking with a 2 M KCl solution (soil: solution ratio: 1:5) for 1 h. After filtering, the extract solution was analyzed using a continuous flow analyzer. Soil NO_3_-N accumulation (SNA) was then calculated using the following formula:

$$\begin{array}{l} \text{SNA}=\text{SNC }\times \;\rho_{\text{b}}\;\times \text{ h} \end{array}$$

Where, ρ_b_ (g cm^−3^) is the soil bulk density, and h (cm) is the soil thickness. The gravimetric water content (θ, g g^−1^) of the samples was determined by drying them to a constant weight in an oven at 105°C (Bao, 2000). Soil water storage (SWS, mm) was calculated as:

$$\begin{array}{l} \text{SWS}=\theta \;\times \;\rho_{\text{b}}\;\times \text{ h} \end{array}$$

In addition, soil water storage reduction amount (SWR) was calculated as the SWS at sowing minus the SWS at maturity for the different soil layers.

The evapotranspiration (ET) of the wheat was calculated by the following formula:

$$\begin{array}{l} \text{ET}=\text{I }+\text{ P }+\text{ Δ}S\;+\;U\;-\;R\;-\;D, \end{array}$$

where ET (mm) is evapotranspiration, I (mm) is the amount of irrigation water that was measured using the water meters, P (mm) is the precipitation that was measured with the weather station at the site shown in Figure 1, ΔS (mm) is the SWS in the soil layer from 0 to 1.6 m at sowing minus the SWS at harvest, R (mm) is surface runoff (there was no surface runoff, so this variable was ignored). U is the upward capillary flow into the root zone (mm), and D is the downward drainage out from the root zone (mm). Because the groundwater table was ~10.2–11.2 m below the soil surface during the 6-year period, capillary flow is negligible (Liu and Wei, 1989). Drainage from the root zone was estimated using the following equation:

$$\begin{array}{l} D=-K\left(\theta \right)\cdot \frac{\Delta \text{H}}{\Delta \text{Z}}\cdot \Delta t \end{array}$$

Where *K* (θ) is the unsaturated water conductivity when soil moisture is θ (cm day^−1^), ΔH is the soil water potential between the top-soil and sub-soil (cm), ΔZ is the soil thickness (cm), and Δ*t* is the period of time (in days). SWC was measured in the 1.6 m soil profile in this study, since more than 90% of the root system of wheat plants occupies only the first 1.0 m soil layer in the North China Plain, as was shown by several previous studies (Zhang et al., 2009; Wang et al., 2014; Xu et al., 2016). Therefore, drainage out from the root zone can be ignored in the North China Plain, including at our experimental site (Lv et al., 2011). Water use efficiency (WUE; kg ha^−1^ mm^−1^) in wheat is defined as grain yield divided by ET over the whole wheat growing season.

### Root sampling

Roots were sampled at maturity by removing soil blocks during the 2013–2014 wheat growing season. Briefly, blocks were dug at 0.2 m intervals to a vertical depth of 1.6 m in six separate layers. Each sampling area was 0.4 m in length (perpendicular to the rows, providing access to plants in two rows) and 0.4 m in width (parallel to the rows). The roots with soil were then transferred to a 100–mesh nylon bag and submerged in water for 1 h. Any remaining soil was then washed from the roots using a low-pressure garden hose, and the clean roots were transferred to a sieve (0.25 mm^2^ mesh) suspended in a trough partially filled with water. The cleaned roots were imaged using a scanner for the gray-scale scanning (Epson perfection V700 photo). The files were then analyzed using WinRHIZO 2008 in order to measure root length. Root length density (RLD; mm cm^−3^), the unit volume of soil root length, was determined using the following formula:

$$\begin{array}{l} \text{RLD}=\text{L}/\text{V}, \end{array}$$

where L is the total root length (mm) and V is the volume of the soil sample (cm^3^).

### Statistical analysis

Statistical analyses were performed using SPSS 17.0 software. Irrigation regime, nitrogen rates, and their interactions were taken as fixed factors, while the growing season was considered to be a random factor due to unpredictable weather conditions. Analysis of variance (ANOVA) was conducted for grain yield, SWS and SNA, ET and WUE, and for SWS, SNC, and RLD at each soil layer. The means of each treatment were compared with Duncan's multiple range test at the 0.05 probability level (at *p* ≤ 0.05). Pearson correlations were used to analyze the relationship between RLD and SWC (*n* = 72) and SNC (*n* = 36), and between yield and water-nitrogen use parameters (*n* = 144). Figures were prepared using Sigmaplot 12.3.

## Results

### Wheat yield

**Figure 2:**
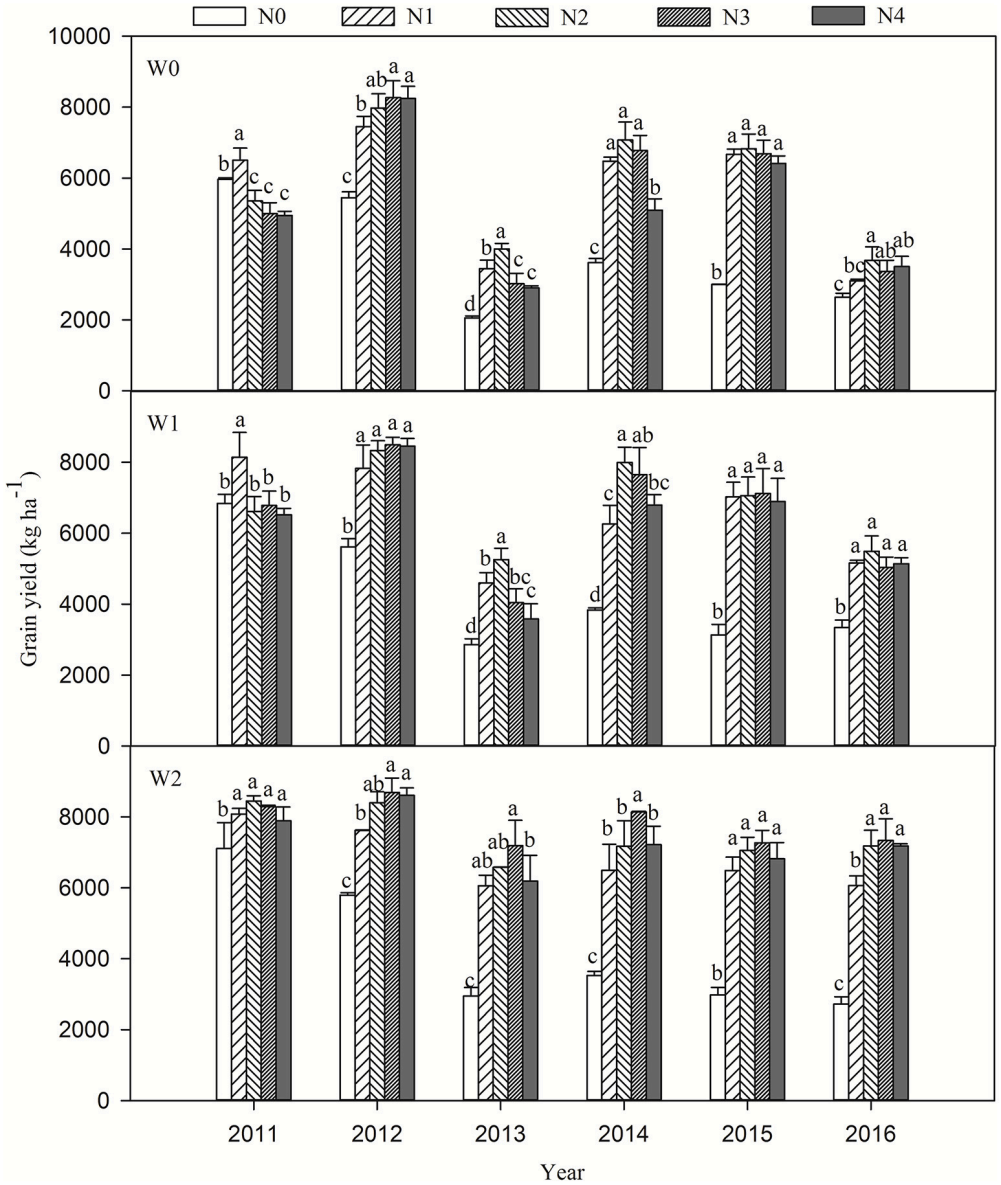
Effects of irrigation and nitrogen application regimes on grain yield in winter wheat from the 2010–2011 to 2015–2016 growing seasons. Different letters above the bars indicate significant differences between wheat yields in the different N treatments at the 5% level under the same irrigation conditions. N0, no applied N; N1, 90 kg ha^−1^; N2, 180 kg ha^−1^; N3, 240 kg ha^−1^; N4, 300 kg ha^−1^.

Wheat yields averaged from 2055 to 8690 kg ha^−1^ in all treatments during the six growing seasons (Figure 2). Compared with the non-irrigated treatment (W0), irrigation at jointing (W1) significantly increased yield by 25.7, 3.6, 31.8, 13.6, 5.6, and 45.6% in the growing seasons from 2010–2011 to 2015–2016, respectively (Figure 2, Table 2). With increased irrigation (i.e., W2), wheat yield was significantly increased by 14.1, 45.3, and 29.3% compared to W1 in the 2010–2011, 2012–2013, and 2013–2016 growing seasons, respectively, in which the rainfall was lower or unevenly distributed during the wheat growing seasons (Figure 1). Correlation analysis (**Table 5**) revealed a strong positive correlation between rainfall and wheat yield (*r* = 0.601^**^). However, no significant differences were found between W1 and W2 in the 2011–2012, 2013–2014, and 2014–2015 growing seasons (Figure 2, Table 2). This indicated that overfull irrigation did not result in higher yields in normal precipitation growing seasons. Wheat yields in irrigated treatments varied less over time compared to non-irrigated treatments, indicating that irrigation not only increased but also stabilized wheat yield. The CV for wheat grain yield in W0 was higher than in the irrigated treatments (W1 and W2), indicating that the precipitation and its distribution may be other important factors affecting wheat yield (Figure 1).

**Table 2:** Mean wheat grain yields for irrigation treatments across N rates and for N rates across irrigation treatments (kg ha^−1^) and *F*-values from analysis of variance for the 2010–2011 to 2015–2016 growing seasons.

**Treatment**	**2010–2011**	**2011–2012**	**2012–2013**	**2013–2014**	**2014–2015**	**2015–2016**	**Average**	**CV (%)**	**% increase**
**IRRIGATION REGIME**									
W0	5552.9c	7394.8b	3025.3c	5726.0b	5800.0b	3237.6c	5122.8c	32.8	
W1	6978.1b	7663.2a	3987.3b	6507.3a	6123.8a	4714.9b	5995.8b	23.2	17.0
W2	7963.7a	7821.5a	5792.9a	6507.7a	6121.7a	6095.8a	6717.2a	14.0	31.1
**N RATE**									
N0	6638.3b	5617.3c	2622.3c	3657.7c	3037.1c	2900.1c	4078.8c	40.6	
N1	7574.8a	7365.1b	4415.5b	6276.7b	6325.7b	4542.1b	6083.3b	22.2	49.1
N2	6804.6b	8234.5a	4925.4a	7411.7a	6979.4a	5298.7a	6609.0a	19.2	62.0
N3	6687.8b	8482.0a	5154.7a	7520.5a	7025.8a	5396.7a	6711.3a	18.9	64.5
N4	6452.4b	8433.6a	4224.6b	6368.3b	6707.8ab	5276.4a	6243.8b	22.7	53.1
Irrigation regimes (W)	167.6^***^	6.6^**^	231.6^***^	18.2^***^	2.5 ^ns^	370.4^***^			
N rate (N)	12.9^***^	122.9^***^	74.5^***^	115.7^***^	158.7^***^	110.5^***^			
Interaction W × N	6.7^***^	0.1^ns^	11.4^***^	6.0^***^	0.4 ^ns^	23.7^***^			

*Wheat yields within a column followed by different letters are significantly different at the 0.05 probability level*.

ns *, indicates no significant difference*.

** *Significant difference at the 0.01 level*.

*** *Significant difference at the 0.001 level*.

N application significantly improved wheat yield, but excessive N rates did not have a positive effect on yield (Figure 2). In the growing seasons from 2010–2011 to 2015–2016, the wheat yield (averaged across irrigation and N treatments) was increased by 3.6, 44.7, 78.5, 88.5, 122.6, and 76.8% in the N treatments compared to the N0 control treatment, respectively (Figure 2, Table 2), suggesting that the effect of N fertilizer on wheat yield became greater as the experimental seasons progressed. Averaged across the six growing seasons, the highest yield of 6711.3 kg ha^−1^ was in the N3 treatment, but there was no significant difference between N2 and N3. Statistical analysis showed that the interaction between irrigation regimes and N rates on yield was significant (Table 2). The W2N3 treatment produced the maximum yield, and the W0N0 treatment gave the lowest yield (Figure 2).

### Soil water storage (SWS)

Maximum SWS (averaged across irrigation and N treatments) before sowing of wheat (388.9 mm) was recorded in the 2011–2012 growing season, whereas the minimum SWS of wheat (274.9 mm) occurred in the 2012–2013 growing season (Table 3), which resulted in the highest and the lowest grain yields in the corresponding seasons (Table 2). No difference in SWS before sowing was observed among the three irrigation regimes in the 2013–2014 and 2014–2015 growing seasons, which resulted in reduced yield increases in the irrigated treatments (Table 2). SWS before sowing in N0 was higher than in the N treatments, and irrigation therefore did not increase yield under no-N conditions. N3 had the lowest SWS before sowing, while W2 gave the highest yield (Figure 2). This suggests that irrigation can increase yield under N application conditions, but that it could not balance the adverse effect of N stress on grain yield under conditions of N deficiency.

**Table 3:** The soil water storage (SWS; mm) and soil NO_3_-N accumulation (SNA; kg ha^−1^) in the 0–1.6 m soil profile prior to sowing winter wheat.

**Parameter**	**SWS**					**SNA**				
	**Oct 7, 2011**	**Oct 5, 2012**	**Oct 6, 2013**	**Oct 4, 2014**	**Oct 5, 2015**	**Oct 7, 2011**	**Oct 5, 2012**	**Oct 6, 2013**	**Oct 4, 2014**	**Oct5, 2015**
**IRRIGATION REGIME**										
W0	388.9	264.2b	298.5a	381.0a	225.0b	241.2	186.3a	191.9a	229.8a	215.5a
W1	388.9	272.5ab	308.1a	371.1a	232.2b	241.2	166.1b	186.8a	160.3b	205.3b
W2	388.9	288.1a	288.5a	364.4a	271.2a	241.2	160.5b	177.1b	142.0c	156.3c
**N RATE**										
N0	388.9	282.9a	289.1a	373.9a	286.9a	241.2	114.2e	141.2d	128.4d	117.9d
N1	388.9	279.5a	289.4a	379.3a	240.3b	241.2	132.2d	159.2c	147.4c	157.9c
N2	388.9	276.9a	291.5a	374.5a	224.7b	241.2	159.0c	170.0c	176.1b	201.7b
N3	388.9	266.2b	310.8a	364.5b	230.9b	241.2	210.4b	195.2b	211.2a	234.5a
N4	388.9	269.2b	310.9a	368.6b	231.1b	241.2	239.0a	261.1a	223.7a	249.8a
Irrigation regimes (W)		20.1^***^	14.9^***^	10.3^***^	16.7 ^***^		23.5^***^	45.2^***^	89.3^***^	47.5^***^
N rate (N)		1.9 ^ns^	17.8^***^	0.3^ns^	30.7^***^		244.4^***^	831.5^***^	43.3^***^	119.2^***^
Interaction W × N		3.7^***^	11.7^***^	2.1^ns^	1.9^ns^		5.4^***^	16.3^***^	14.4^***^	7.6^***^

*Numbers within a column followed by different letters are significantly different at the 0.05 probability level. ns, indicates no significant difference*.

*** *Significant difference at the 0.001 level*.

**Figure 3:**
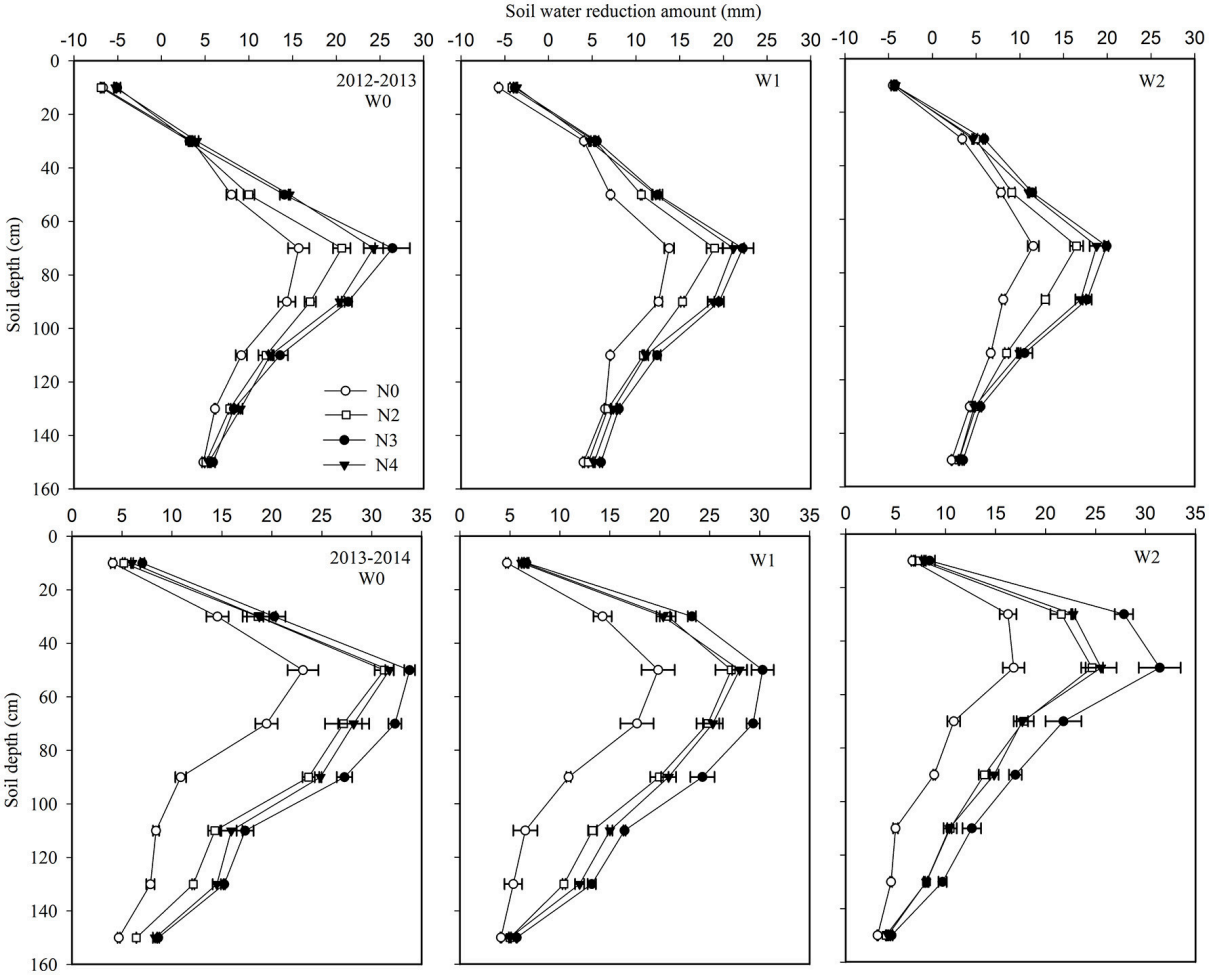
Effects of irrigation and nitrogen regimes on soil water reduction amount at 0.2 m soil depth increments to 1.6 m in the 2012–2013 and 2013–2014 growing seasons. Horizontal bars represent the standard errors.

Plots of the soil water reduction amount (SWR) in the different soil layers are shown in Figure 3. Water was mainly consumed in the 0.4–1.2 m deep layers, with the highest SWR in the 0.6–1.0 m layer. The proportion of SWR in the 1.0–1.6 m layer was higher in the drier growing season 2012–2013 (32.0%) than in the normal precipitation growing season 2013–2014 (22.4%). Similarly, SWR in W0 was higher than in W1 and W2 at all soil layers (Figure 3). N rates had a significant effect on SWR, since N application increased SWR in all soil layers. The N3 treatment gave the highest SWR (Figure 3). Moderate water applied (W1) and appropriate N rates facilitated longer root growth, which resulted in more water being consumed in the deeper soil layers.

### ET and WUE

Irrigation caused a marked increase in evapotranspiration (ET). Compared to the un-irrigated treatments, ET in the W2 treatment increased by 45.2% during the drier growing seasons (averaged across 2012–2013 and 2015–2016), but only increased by 18.6% during growing seasons with normal precipitation (averaged across 2013–2014 and 2014–2015; Table 3). The W2 treatment showed a significant reduction in WUE in the 2013–2014 and 2014–2015 growing seasons, but gave the maximum WUE values during the 2012–2013 and 2015–2016 growing seasons (Table 4), which might be attributed to less rainfall and uneven distribution (Figure 1). N fertilizer caused an increase in ET that averaged 32.5, 38.2, and 34.5 mm for the N180, N240, and N300 treatments compared to N0, respectively (Table 3). Compared to the N0 treatment, N treatment significantly increased WUE by 76.4, 69.0, 96.1, and 70.9% (averaged across irrigation and N treatments) for the 2012–2013 to 2015–2016 growing seasons, respectively (Table 4). However, there was no further increase in WUE when the nitrogen application rate exceeded 180 kg N ha^−1^.

**Table 4:** Effects of irrigation and nitrogen regimes on evapotranspiration (ET; mm) and water use efficiency (WUE; kg ha^−1^ mm^−1^) of winter wheat for the 2012–2013 to 2015–2016 growing seasons.

**Treatment**		**ET**				**WUE**			
		**2012–2013**	**2013–2014**	**2014–2015**	**2015–2016**	**2012–2013**	**2013–2014**	**2014–2015**	**2015–2016**
W0	N0	241.5b	369.3c	412.8b	261.4c	8.5c	9.8c	7.3b	10.1c
	N2	248.4a	410.8b	481.1a	267.4b	16.1a	17.2a	14.2a	13.8a
	N3	234.6b	422.2a	480.2a	278.5a	12.9b	16.0a	13.9a	12.1b
	N4	243.5b	421.2a	480.1a	277.0a	11.9b	12.1b	13.4a	12.7b
W1	N0	326.1b	442.3c	459.8c	338.4b	8.8d	8.7d	6.8b	9.9c
	N2	337.6a	478.2b	534.4a	346.3a	12.4b	16.7a	13.2a	14.5a
	N3	325.2b	518.6a	528.7a	349.0a	16.2a	14.8b	13.5a	15.7a
	N4	323.4b	506.8a	514.3b	341.2ab	11.1c	13.4c	13.4a	15.1a
W2	N0	397.1a	473.3c	470.6c	415.5ab	7.4c	7.4b	6.3c	6.5b
	N2	401.8a	531.4b	537.5b	423.6a	16.4ab	13.5a	13.1a	16.9a
	N3	398.3a	564.4a	545.3ab	421.6a	18.1a	14.4a	13.3a	17.4a
	N4	396.4a	544.6ab	568.7a	405.1b	15.6b	13.2a	12.0b	17.7a
Irrigation regimes (W)	8374.9^***^	987.8^***^	211.6^***^	3208.4 ^***^	26.9^***^	2.1^ns^	5.1^**^	56.8^***^	
N rate (N)	2028.9^***^	46.6^***^	15.0^***^	395.9 ^***^	11.5^***^	42.0^***^	34.8^***^	22.8^***^	
Interaction W × N	172.6^***^	25.3^***^	30.2^***^	62.7^***^	40.9^***^	40.8^***^	72.6^***^	38.2^***^	

*Numbers within a column followed by different letters under the same irrigation conditions are significantly different at the 0.05 probability level. ns, indicates no significant difference*.

** *Significant difference at the 0.01 level*.

*** *Significant difference at the 0.001 level*.

### Soil NO_3_-N content (SNC)

**Figure 4:**
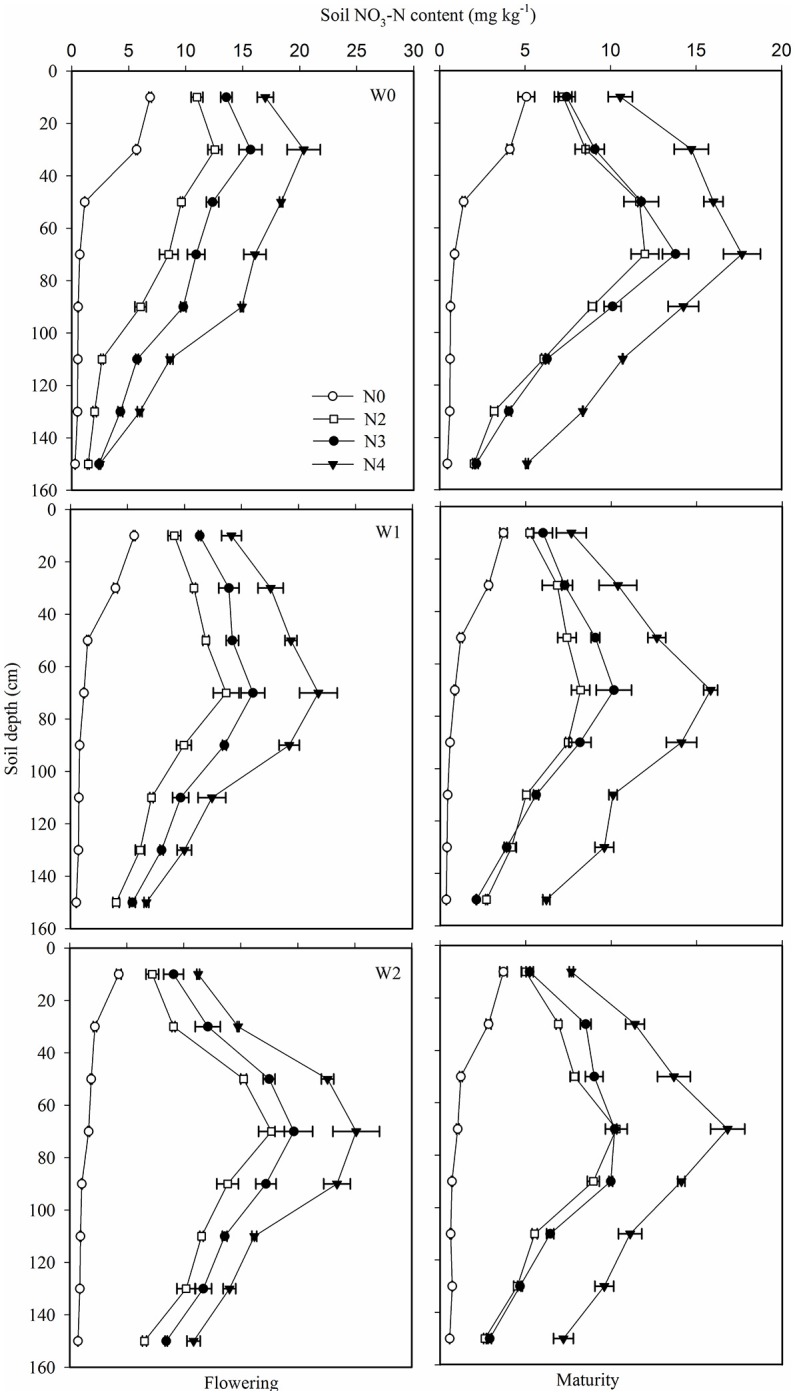
Effect of irrigation and nitrogen regimes on NO_3_-N content at flowering **(left panels)** and maturity **(right panels)** at 0.2 m increments to a depth of 1.6 m in the 2013–2014 growing season. Horizontal bars represent the standard errors.

W2 gave the lowest soil NO_3_-N content (SNC), while W0 had the highest SNC in the top 0.4 m soil layer. However, from 0.8 m downwards, SNC in the irrigated treatments was greater than in the W0 control (Figure 4). This suggests that irrigation increased the levels of residual NO_3_-N in the deep soil layers. Compared to N0, all N treatments significantly increased SNC in the 0–1.6 m soil layer at both flowering and maturity (Table 3, Figure 4). SNC in the N3 treatment was higher than in N2 in the 0.6–1.4 m soil layers at flowering, while no difference was found between N2 and N3 at maturity, indicating that N3 improved absorption of NO_3_-N in the 0.6–1.4 m soil layers during the later stages of growth. This might be attributed to plants in the N3 treatment having the most extensive root systems in the deep soil layers. SNC in the 0.8–1.6 m soil layers in the N4 treatment was 37.7 and 86.9% higher than in N3 at flowering and maturity, respectively (Figure 2), implying that there was more residual NO_3_-N in the deep soil layer in N4, which exceeded the ability of wheat to absorb it. Table 3 also shows that the soil nitrogen supply in N0 gradually declined as the experimental seasons progressed, which was in agreement with the slight decrease observed in grain yield in the N0 treatment (Figure 2).

### Root length density (RLD)

**Figure 5:**
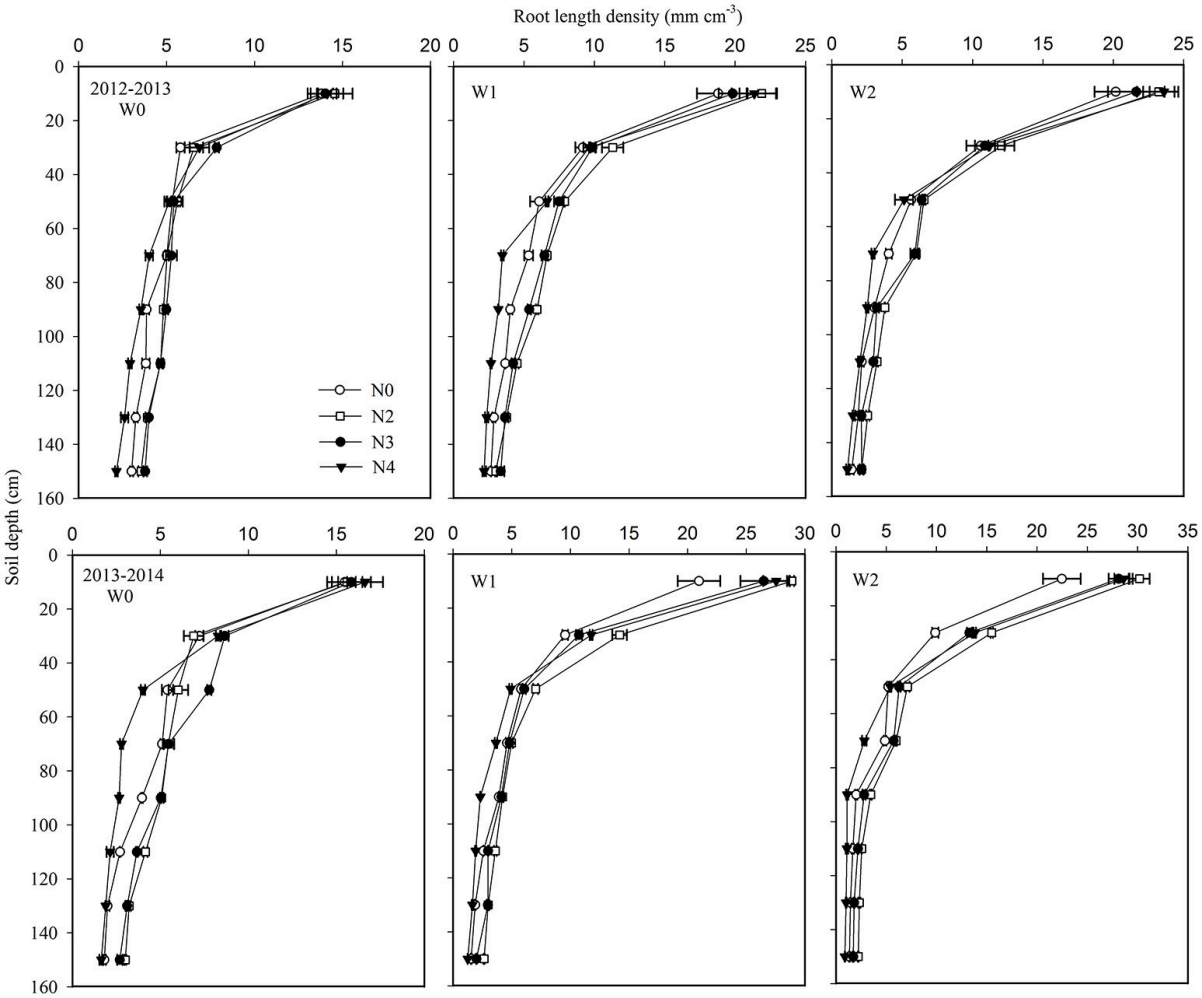
Effects of irrigation and nitrogen regimes on root distribution at 0.2 m depth increments to 1.6 m at maturity in the 2012–2013 and 2013–2014 growing seasons. Horizontal bars represent the standard errors.

Root length density (RLD) declined with increasing soil depth. Averaged across the irrigation and N treatments and both growing seasons, the highest RLD was 21.0 mm cm^−3^ in the 0–0.2 m soil layer, gradually decreasing to 10.1, 6.0, 4.9, 3.7, 3.0, 2.5, and 2.2 mm cm^−3^ at 0.2 m intervals through the soil column to a depth of 1.6 m. The W2 treatment had the highest RLD, while W0 plants had the smallest root system in the top 0.4 m soil layer (Figure 5). The RLD in the W0 and W1 treatments was greater than in W2 from 0.8 m downwards. The N application rates of 180 and 240 kg ha^−1^ (N2 and N3) gave higher RLD values at depths from 0.4 to 1.6 m, while a higher RLD was only found in the 0–0.4 m soil layer in the N4 treatment. The relationships between RLD distribution and SWR and SNC for the different soil profiles were analyzed at 0.4-m intervals (**Table 6**). Positive relationships between RLD and SWR, and RLD and SNC were found in each soil layer for the non-irrigated and no-N treatments. SWC and SNC at soil depths of 0–0.4 and 0.8–1.6 m exhibited significant positive correlations with RLD for the W1 and N2 treatments, while a significant correlation was only found in the upper soil profile (0–0.4 m) for the W2 and N4 treatments. This suggests that elevated water and N use in deep soil layers under optimum irrigation and N regimes can be attributed to a deeper root system.

## Discussion

When the crop growing season was dry, irrigation was necessary in order to offset a water deficit. It has been previously reported that supplemental irrigation and limited or deficit irrigation greatly affects both crop yield and WUE (Zhang et al., 1999; Wang et al., 2001; Sun et al., 2010). Compared to rainfed conditions, grain yield with supplemental irrigation was significantly increased by 8.0% in the North China Plain (Li et al., 2015), and by 25–35% in the Lake Chad Basin of Africa (Karam et al., 2009). Optimal irrigation levels and mild soil water stress conditions (65–70% of field capacity) significantly increased yield and WUE in wheat (Zhang et al., 2003; Liu et al., 2016). However, some studies have indicated that deficit irrigation significantly decreased wheat yield (Adelian et al., 2012; Chen et al., 2015). In the present study, compared to the W0 control treatment, grain yield increased by 3.6–45.6% in W1 and by 5.8–91.5% in W2. No significant difference was found between the W1 and W2 treatments during normal precipitation growing seasons, in which the SWS before sowing was relatively high. Correlation analysis showed a significant positive correlation between SWS before sowing and grain yield (Table 5), which might be attributed to the precipitation and irrigation during the maize growing seasons. In the high SWS growing seasons of 2011 and 2014, the September rainfall was 260.0 and 212.3 mm, respectively (Figure 1). In 2013, although there was less rainfall in September, irrigation was applied during the late growing stage of maize (Table 1). This suggested that, for wheat production, it is critical to make use of additional water from summer maize. In the experimental area, precipitation was sufficient to meet the needs of maize production, and irrigation usually had no beneficial effect on maize yield (data not shown). Correlation analysis showed no significant correlation between precipitation and maize yield, but wheat yield was significantly influenced by precipitation (*r* = 0.601^**^). During the drier growing seasons, the relative increase in wheat yield was higher (54.4%) than that during normal precipitation growing seasons (8.0%). High soil moisture content throughout the seasons resulted in high levels of water consumption but did not lead to higher grain yields (Kang et al., 2002; Chen et al., 2015). Qiu et al. (2008) suggested that high grain yield in wheat can be obtained by no- or one-time irrigation in the North China Plain. Our results showed that irrigation at the wheat jointing stage can produce high grain yields with a sufficient pre-sowing soil moisture level. With increases in water (i.e., W2), ET increased, but grain yield did not increase. WUE decreased significantly during normal precipitation growing seasons. But during the drier growing seasons, grain yield, ET, and WUE increased simultaneously. Growers can make decisions for the second irrigation (from booting to flowering) based on precipitation, soil water content and wheat growth. These informations can be obtained from local service centers of agricultural technology in China.

**Table 5:** Pearson correlations of soil water storage (SWS) before sowing and soil NO_3_-N accumulation (SNA) before sowing with rainfall, yield, and water use efficiency (WUE) in winter wheat.

**Items**	**Rainfall**	**Yield**	**SWS**	**WUE**	**SNA**
Rainfall	1				
Yield	0.601^**^	1			
SWS	0.680^**^	0.384^**^	1		
WUE	0.179	0.790^**^	0.294^*^	1	
SNA	0.621^**^	0.366^**^	0.401^**^	0.311^**^	1

* *Represents significance at P < 0.05*.

** *Represents significance at P < 0.01*.

N fertilizer is the basis of high yield (Wang et al., 2011; Sinclair and Rufty, 2012) and WUE (Shangguan et al., 2000; Brueck and Senbayram, 2009). Grain yield and WUE both increased at N application rates of 200–300 kg N ha^−1^ under sufficient soil water conditions (Sepaskhah and Tafteh, 2012). Deng et al. (2006) found that N fertilizer increased wheat yield by 71.3% and WUE by 20.1% in north and northwest China. In the present study, compared to N0, the N treatments increased grain yield by 49.1–64.5% and WUE by 66.9–83.9%, respectively. However, there was no further increase in grain yield or WUE when N rates exceeded 240 and 180 kg N ha^−1^, respectively. Excessive N application and irrigation resulted in high residual soil NO_3_-N after the wheat harvest (Wang et al., 2010, 2015), and 172 kg N ha^−1^ at a soil depth of 0.9 m was previously observed under high N fertilizer conditions in the North China Plain (Cui et al., 2010). After 6 years of N application, there was about twice as much SNA at sowing in the soil profile in the N4 treatment as in the N0 control, but there was ~27.5% less in the W2 treatment than in W0 control (Table 3). The distribution of soil NO_3_-N was also determined by the movement of soil water, and followed with water infiltration (Perego et al., 2012; Sharma et al., 2012). This indicated that NO_3_-N leached out of the 1.0 m soil layer in the wheat growing seasons with each irrigation amount of 75 mm (Liu et al., 2005), and leached out of the 2.0 m soil layer when each irrigation amount was 100 mm (Gu et al., 2015). In the present study, SWR declined from 0.8 m downwards due to high SWC below 0.8 m soil depth at harvest, and the peaks of SNA occurred in the 0.6–0.8 m soil layer after 3 years.

Root distribution is essential for nutrient and moisture absorption, and therefore, growth and final yield in crops (Zhang et al., 2009; Li et al., 2010). Roots in the deep soil profile (>0.8 m) play an important role in the utilization of soil water after flowering in wheat (Li et al., 2010; Xu et al., 2016) and, therefore, increasing WUE (Zhang et al., 2004). In the present study, a significant positive relationship was found between RLD and both SWR and SNC in the single irrigation treatment at jointing combined with N application rates of 180–240 kg N ha^−1^ in both upper and deep soil layers, which confirmed that wheat not only utilizes soil water and N from the upper soil layers but also from the deep layers. With late irrigation and N application (after the flowering stage), less irrigation water and N are used by wheat (Kang et al., 2000; Li et al., 2010). Under the sufficient water (W2) and excessive N supplementation (N4) treatments, water and N were not limiting factors, reducing the need for roots to absorb water and N from deeper soil layers (Table 6). These findings indicate that appropriate N application (180–240 kg ha^−1^) with a single irrigation at jointing can improve the ability of wheat to absorb soil water and N in the 0.6–1.4 m soil layer during the growing season, which might be attributed to the more extensive root system in the deeper soil layers.

**Table 6:** Correlation coefficients between root length density (RLD) and soil water consumption (SWC) and soil NO_3_-N content (SNC) at different soil depths.

**Item**	**RLD in the W0 treatment**				**RLD in the W1 treatment**				**RLD in the W2 treatment**			
**IRRIGATION REGIME**												
Soil depth (m)	0–0.4	0.4–0.8	0.8–1.2	1.2–1.6	0–0.4	0.4–0.8	0.8–1.2	1.2–1.6	0–0.4	0.4–0.8	0.8–1.2	1.2–1.6
SWC	0.372^*^	0.483^*^	0.661^**^	0.557^**^	0.346^*^	0.079	0.360^*^	0.535^**^	0.731^**^	0.234	0.101	0.021
SNC	0.790^**^	0.824^**^	0.883^**^	0.875^**^	0.843^**^	0.512	0.241	0.769^**^	0.874^**^	0.389	0.313	0.213
**N RATE**												
	**RLD in the N0 treatment**				**RLD in the N2 treatment**				**RLD in the N4 treatment**			
Soil depth (m)	0–0.4	0.4–0.8	0.8–1.2	1.2–1.6	0–0.4	0.4–0.8	0.8–1.2	1.2–1.6	0–0.4	0.4–0.8	0.8–1.2	1.2–1.6
SWC	0.379^*^	0.410^*^	0.810^**^	0.671^**^	0.483^*^	0.111	0.399^*^	0.761^**^	0.824^**^	0.200	0.098	0.044
SNC	0.651^**^	0.789^**^	0.851^**^	0.856^**^	0.765^**^	0.223	0.654^**^	0.521^*^	0.912^**^	0.414	0.365	0.216

* *Represents significance at P < 0.05*.

** *Represents significance at P < 0.01*.

The *F*-value gives an indication of statistical significance, and the size of the effect of the treatments can be obtained from the variables measured (Wang et al., 2010). Our results show that N fertilizer rates had higher *F*-values than irrigation regimes during normal precipitation growing seasons, but irrigation regimes had higher *F*-values than did N rates during the drier growing seasons. This might be attributed to the relatively high SNA before sowing in the 1.6 m soil profile (from 148.6 to 243.0 kg N ha^−1^). Correlation analyses also showed that grain yield and WUE were significantly influenced by SNA before sowing. Abad et al. (2004) found that if the starting SNA in the 0–0.9 m soil profile was < 157 kg N ha^−1^, the yield would increase clearly with N applications up to 100 kg N ha^−1^ and high initial SNA reduced the response of yield to N rates.

Adequate water supply increases the availability of N fertilizer, while an adequate N fertilizer supply is a benefit to utilizing additional water from summer maize rainfall (Tilling et al., 2007; Di Paolo and Rinaldi, 2008; Sadras et al., 2012; Wang et al., 2015). Guo et al. (2012) concluded that the optimum N application rates in dry, normal, and wet years were 45, 135, and 180 kg ha^−1^, respectively, in a rainfed wheat cropping system in the semiarid Loess Plateau of China. Under well-water conditions, yield and WUE are enhanced under adequate N application rates of 200–300 kg N ha^−1^ (Sinclair and Rufty, 2012). Under current Huang-Huai climate conditions, application of N at rates of 180 and 240 kg N ha^−1^ produced the maximum grain yield under non-irrigated and irrigated conditions, respectively. The appropriate irrigation (a single irrigation at jointing) and N application rates (180–240 kg ha^−1^) favorably promoted the use of soil water and NO_3_-N mainly by increasing the number of roots in the deep soil layers, which is conducive to achieving high WUE with relatively high grain yield.

## Conclusions

Our experiments revealed that N is the predominant factor that limits grain yield during normal precipitation growing seasons. During the drier growing seasons, irrigation had a relatively larger effect on wheat yield. Irrigation at the jointing stage gave higher yields and WUE during normal precipitation growing seasons. However, an additional irrigation at flowering (W2) was necessary in the drier growing seasons. Appropriate N application rates facilitated root development, which not only resulted in higher yields and WUE, but also reduced residual NO_3_-N. When irrigation was combined with N rates of 180–240 kg N ha^−1^, it favorably enhanced the use of soil water and NO_3_-N in both the upper and deeper soil layers by regulating root growth, which is conducive to attaining high grain yields and WUE.

## Author contributions

CW: conceived of and designed the study; WL and GM: analyzed the data and wrote the manuscript; WL, SL, and JW: carried out the field measurements and root analysis; HL, WF, and YX: critically reviewed the manuscript; DM and GK: assisted with manuscript writing and editing. All authors approved the final version of the manuscript.

### Conflict of interest statement

The authors declare that the research was conducted in the absence of any commercial or financial relationships that could be construed as a potential conflict of interest.
